# Extracellular Vesicles From *Paracoccidioides brasiliensis* Can Induce the Expression of Fungal Virulence Traits *In Vitro* and Enhance Infection in Mice

**DOI:** 10.3389/fcimb.2022.834653

**Published:** 2022-02-28

**Authors:** Carla Elizabete Octaviano, Nadiellen E. Abrantes, Rosana Puccia

**Affiliations:** Departamento de Microbiologia, Imunologia e Parasitologia, Escola Paulista de Medicina-Universidade Federal de São Paulo (EPM-UNIFESP), São Paulo, Brazil

**Keywords:** *Paracoccidioides brasiliensis*, virulent/attenuated Pb18, extracellular vesicles, virulence transference, stress

## Abstract

Extracellular vesicles (EVs) are cellular components involved in cargo delivery to the extracellular environment, including the fungal cell wall. Their importance in cell–cell communication, cell wall remodeling, and fungal virulence is starting to be better explored. In the human pathogenic *Paracoccidioides* spp., our group has pioneered the description of the EV secretome, carbohydrate cargo, surface oligosaccharide ligands, lipid, and RNA content. Presently, we studied the role of fungal EVs in the context of the virulent/attenuated model of the *P. brasiliensis* Pb18 isolate, which consists of variants transiently displaying higher (vPb18) or attenuated (aPb18) virulence capacity. In this model, the virulence traits can be recovered through passages of aPb18 in mice. Here, we have been able to revert the aPb18 sensitivity to growth under oxidative and nitrosative stress upon previous co-incubation with vEVs from virulent vPb18. That was probably due to the expression of antioxidant molecules, considering that we observed increased gene expression of the alternative oxidase *AOX* and peroxiredoxins *HYR1* and *PRX1*, in addition to higher catalase activity. We showed that aEVs from aPb18 stimulated macrophages of the RAW 264.7 and bone marrow-derived types to express high levels of inflammatory mediators, specifically, TNF-α, IL-6, MCP-1, and NO. In our experimental conditions, subcutaneous treatment with EVs (three doses, 7-day intervals) before vPb18 challenge exacerbated murine PCM, as concluded by higher colony-forming units in the lungs after 30 days of infection and histopathology analysis. That effect was largely pronounced after treatment with aEVs, probably because the lung TNF-α, IFN-γ, IL-6, and MCP-1 concentrations were specially increased in aEV-treated when compared with vEV-treated mice. Our present studies were performed with EVs isolated from yeast cell washes of confluent cultures in Ham’s F-12 defined medium. Under these conditions, vEVs and aEVs have similar sizes but probably distinct cargo, considering that vEVs tended to aggregate upon storage at 4°C and −20°C. Additionally, aEVs have decreased amounts of carbohydrate and protein. Our work brings important contribution to the understanding of the role of fungal EVs in cell–cell communication and on the effect of EVs in fungal infection, which clearly depends on the experimental conditions because EVs are complex and dynamic structures.

## Introduction

Human paracoccidioidomycosis (PCM) is currently defined as a systemic mycosis caused by isolates of the *Paracoccidioides brasiliensis* complex, specifically *P. brasiliensis sensu strictu* (S1 main group), *P. americana* (PS2 group), *P. restrepiensis* (PS3 group), and *P. venezuelensis* (PS4 group), and by *Paracoccidioides lutzii*, formerly referred to as “Pb01-like” (Matute et al., 2006; Teixeira et al., 2014; Turissini et al., 2017). We have observed that isolates of phylogenetic group S1 are more virulent than those from PS2 using the intratracheal PCM mice model, thus suggesting a correlation between genetic groups and virulence (Carvalho et al., 2005). *Paracoccidioides* spp. are temperature-dependent dimorphic ascomycetes that grow as mycelia at temperatures up to 28°C and as multibudding, multinucleated yeasts at incubation temperatures around 37°C or in the host tissues (Teixeira et al., 2014).

PCM is a granulomatous mycosis that affects mainly the lungs (Martinez, 2017). Protection against PCM is provided by Th1-driven pro-inflammatory immune responses initiated by dendritic cells expressing interleukin (IL)-12 (Gow et al., 2017; Burger, 2021; Fernández-García and Cuellar-Rodríguez, 2021). Active macrophages and neutrophils are detrimental to the prevention of disease progression. Interferon γ (IFN-γ) activates macrophages to express tumor necrosis factor-α (TNF-α) that supports granuloma persistence. The production of effector molecules such as reactive oxygen (ROS) and nitrogen (NOS) species of the respiratory burst by the immune cells is a key effector mechanism in fungal clearance (Gow et al., 2017; Burger, 2021; Fernández-García and Cuellar-Rodríguez, 2021).

Since their characterization in *Cryptococcus neoformans* (Rodrigues et al., 2007), extracellular vesicles (EVs) have been recognized as important fungal components involved in the delivery of cargo to the cell wall and extracellular environment (Zamith-Miranda et al., 2018). The fungal EV cargo is numerous and diverse, comprising membrane lipids, hundreds of proteins (including active enzymes), peptides, small and messenger RNA species, polysaccharides, oligosaccharides, and pigment. The importance of fungal EVs in cell–cell communication, cell wall remodeling, and virulence has recently started to be better explored (Joffe et al., 2016; Nimrichter et al., 2016; Zamith-Miranda et al., 2018a; Zamith-Miranda et al., 2021a; Zamith-Miranda et al., 2021b). Early interest in studying the *Paracoccidioides* EV content resulted in a series of publications by our group and collaborators that described the EV secretome, carbohydrate cargo, surface oligosaccharide ligands, lipid, and RNA content (Vallejo et al., 2011; Vallejo et al., 2012a; Vallejo et al., 2012b; Longo et al., 2014; da Silva et al., 2015a; da Silva et al., 2015b; Peres da Silva et al., 2019; reviewed in Puccia, 2021). These publications have provided not only detailed description of the EV cargo in isolates representing different *Paracoccidioides* species, but also a thorough comparison with published fungal cell wall and EV components. We have especially focused our studies on the Pb18 isolate, which has broadly been used by the PCM community because it is highly virulent in mice (Calich et al., 1985).

The role of *P. brasiliensis* EVs has so far been demonstrated in two publications, both of them using virulent Pb18 as a model. The work by da Silva et al. (2016) showed that Pb18 EVs stimulate the expression of pro-inflammatory mediators in murine peritoneal macrophages, specifically NO, IL-12p40, IL-12p70, IL-6, TNF-α, IL-1a, and IL-1b, and in culture J774A.1 macrophage, specifically IL-12, IL-6, and TNF-α. The Pb18 EVs also stimulated peritoneal macrophage polarization to the M1 phenotype, were able to revert the macrophage phenotype from M2 to M1, and stimulated high macrophage fungicidal activity. More recently, Baltazar et al. (2021) managed to protect mice against PCM upon inoculation of Pb18 EVs previously to fungal challenge.

*Paracoccidioides* spp. still lack an efficient molecular strategy for targeted gene inactivation (Chaves et al., 2021) and studies on the biological role of individual genes are based on *Agrobacterium tumefaciens*-mediated transformation in conjunction with the anti-sense RNA technology to obtain mutants with low expression of the target genes (Sturme et al., 2011; Menino et al., 2012). Virulent *P. brasiliensis* samples kept in the laboratory through continuous subculturing in culture media tend to become attenuated in animal models, but are able to revert to their original pathogenic capacity upon one or more passages in mice and fungal recovery from infected organs (San-Blas et al., 1977; San-Blas et al., 1984; Zacharias et al., 1986; Castaneda et al., 1987; Brummer et al., 1990; Kashino et al., 1990). The virulent/attenuated Pb18 system has been used to screen for virulence factors (Castilho et al., 2014; Castilho et al., 2018). Comparative proteome analysis from virulent/attenuated Pb18 revealed that enzymes related to oxidative stress were more abundant in the virulent Pb18 than in the attenuated Pb18 variant (Castilho et al., 2014). In addition, the enzyme activity of catalase, dismutase superoxide (SOD), and peroxiredoxin Prx1 and the transcription of genes of antioxidant proteins were generally higher in the originally virulent Pb18 than in the attenuated variant (Castilho et al., 2018). The attenuated Pb18 was more sensitive to oxidative stress; however, this trait was reverted after two passages in mice.

A detailed comparison of the EV proteome from *P. brasiliensis* Pb18 with EV proteomes from *C. neoformans*, *Histoplasma capsulatum*, *Candida albicans*, and *Saccharomyces cerevisiae* revealed a 63% overlap among EV orthologs and 26 of them were common to all systems (Vallejo et al., 2012b). Among the common EV proteins are enzymes involved in the fungal defense against oxidative stress, specifically SOD, thioredoxin, and peroxiredoxin Prx1. In addition, the number of EV protein sequences considerably increased when produced under mild oxidative and nitrosative stress conditions, as recently demonstrated for *P. brasiliensis* in our laboratory (Leitão, 2017).

In the present work, we used the virulent/attenuated Pb18 model to evaluate the capacity of EVs to induce the expression of virulence traits in the attenuated variant and to compare their capacity to stimulate immune responses and protect against murine PCM.

## Materials and Methods

### Fungal Strains and Culture Conditions

Our work was carried out with the yeast phase from the *P. brasiliensis* isolate Pb18, whose virulence has been maintained in the lab over the years through mouse infection and recovery from the lungs in brain heart infusion (BHI) agar medium (Taborda et al., 1998). This isolate has been designated vPb18 in the present work to differentiate from its attenuated variant aPb18, which has been obtained after continuous subculturing in modified mYPD (0.5% yeast extract, 1% casein peptone, 0.5% glucose, pH 6.5) agar for years (Castilho et al., 2014). The vPb18 isolate recovered from mice lungs in BHI was subcultured in mYPD slants for 7 days at 36°C and kept at 4°C for up to 3 months. The aPb18 variant was also kept medium term at 4°C. At the appropriate time, the stored yeasts were recovered in fresh mYPD agar at 36°C for 6–7 days and seeded in 200 ml of defined Ham’s F-12 medium (Life Technologies, Grand Island, NY, USA) supplemented with 0.5% glucose (Ham/glc), under agitation (120 rpm) for 4 days to obtain a log-phase pre-inoculum that was used in the following experiments.

### Animals

Balb/C male mice (6 to 8 weeks old) were purchased from CEDEME (Centro de Desenvolvimento de Modelos Experimentais, UNIFESP) and maintained in sterilized cages at 23°C to 25°C under 12-h cycles of light/dark. The animal procedures followed the ethical handling of laboratory animals approved by the UNIFESP Animal Experimentation Ethics Committee (CEUA/UNIFESP), under protocol number 8450221018.

### EV Preparation

We adapted for *Paracoccidioides* the protocol recently reported for *C. neoformans* using fungal washes of confluent growth scrapped from agar plates (Reis et al., 2019) to isolate EVs. Briefly, yeast cells from a 4-day, logarithmic growing pre-inoculum in Ham/glc were pelleted by centrifugation and counted for cell viability in Trypan blue. A total of 9 × 10^6^ viable cells/600 µl/plate were spread in Petri dishes (90 × 15 mm) containing Ham/glc agar and incubated for 2 days at 36°C, when they started to reach confluent growth at >95% viable cells. For one EV preparation, the growth from three Petri dishes was gently scrapped and transferred to a conic tube containing 30 ml of sterile PBS and the cells were precipitated by centrifugation (4,000×*g* for 15 min at 4°C). The supernatant was cleared of cell debris for 30 min at 15,000×*g*, filtered through a 0.45-μm membrane and ultracentrifuged at 100,000×*g* for 1 h at 4°C (Vallejo et al., 2011; Reis et al., 2019). The pellet was washed in PBS and suspended in 300 ml PBS (phosphate-buffered saline). We denominated vEVs and aEVs as the preparations derived from, respectively, vPb18 (virulent) and aPb18 (attenuated variant). Confluent growth in mYPD was achieved with spreading cells from a mYPD pre-inoculum.

### EV Size Characterization and Sterol and Protein Contents

The diameter and concentration of EV preparations were estimated by nanoparticle-tracking analysis (NTA). The preparations were diluted 50 times in sterile PBS and analyzed in a NanoSight NS300 (Malvern Instruments Ltd., Worcestershire, UK) after 5 captures/60 s each. The images were interpreted using the NTA software, 3.2 version (Malvern Instruments Ltd., Worcestershire, UK). The sterol content of EV preparations was estimated using the Amplex™ Red Cholesterol Assay Kit (Invitrogen™, Carlsbad, CA, United States), while the protein content was estimated using the Pierce™ BCA Protein Assay Kit (Thermo Scientific™, Rockford, Illinois, USA), following the manufacturer’s instructions.

### EV Co-Incubation Assays With Pb18 Yeast Cells—Viability Tests

The effect of vEV in the aPb18 phenotype was assayed after cocultures in 24-well plates (1 ml Ham-glc/well) of 1.5 × 10^8^ viable aPb18 yeasts/ml with freshly prepared vEVs (100 EVs:1 cell or 1.5 × 10^10^ EVs/ml) for 4 h at 36°C, under agitation (170 rpm). The cells used in the assay were collected from a 4-day log-phase pre-inoculum in Ham/glc. Co-incubated cells were i) tested for viability under oxidative and nitrosative stress and ii) submitted to oxidative stress for the evaluation of gene expression and catalase enzymatic activity, as detailed further. Viability tests were carried out in serially diluted 10-µl spots (1, 10, 50, and 100×) in mYPD agar supplemented with increasing concentrations of either H_2_O_2_ (0, 0.1, 1, 5, 10 mM) or NaNO_2_ (0, 0.25, 0.5, 1 µM). The results were recorded as pictures after 7 days of growth at 36°C. The assays were performed in triplicates.

### RNA Extraction and qPCR Analysis of Stress-Related Genes

aPb18 yeast cells, co-incubated or not with vEVs, and control vPb18 were submitted to oxidative stress in Ham/glc supplemented with 10 mM H_2_O_2_ for 2 h at 36°C, under agitation (170 rpm) in 24-well plates. For RNA extraction, cell pellets were mechanically disrupted by vortexing in TRIzol reagent (Thermo Fisher Scientific, Waltham, MA, USA) with similar volume of glass beads for 10 min (Rocha et al., 2009). Total RNA was isolated from TRIzol cell extracts following chloroform extraction and isopropanol precipitation. Genomic DNA was removed using the TURBO DNA-free™ kit (Thermo Fisher). DNA-free total RNA was quantified in NanoDrop (Thermo Scientific™, Rockford, Illinois, USA) and kept aliquoted at −80°C until use. The SuperScript™ First-Strand Synthesis System kit (Invitrogen™, Carlsbad, CA, United States) was used to obtain cDNA from reverse-transcribed total RNA (1 µg) and oligo(dT)_12–18_ primers. Quantitative PCR (qPCR) was then performed in triplicate using a SYBR-green-based PCR master mix (Applied Biosystems™, Foster City, CA, USA), reverse-transcribed cDNA template (100 ng), and 100 ng of each primer (**Table 1**) in 20 µl final volumes. Cycling was carried out in a Real-Time 7500 thermocycler (StepOnePlus™ Real-Time PCR System—Applied Biosystems™), starting with holding stages at 50°C (10 min) and 95°C (5 min), followed by 40 cycles alternating 95°C (30 s) and 60°C (60 s). The dissociation curve was determined from an additional cycle of 95°C (15 s), 60°C (60 s), and 95°C (15 s). The relative transcript levels were calculated following the threshold 2−ΔΔCT cycle method (Schmittgen and Livak, 2008). The normalization of cycle thresholds was based on the expression of the alpha-tubulin gene (XM_010765319.1), which is suitable for *P. brasiliensis* qPCR because its expression does not tend to fluctuate under oxidative stress (de Arruda Grossklaus et al., 2013).

**Table 1:** Primer pairs used in qPCR.

Gene	Sequence
α-Tubulin (*TUB*)	Sense 5′ CGGCTAATGGAAAATACATGGC 3′
	Anti-sense 5′ GTCTTGGCCTTGAGAGATGCAA 3′
Alternative oxidase (*AOX*)	Sense 5′ AGGGCTGGGAAATATTCTTTG 3′ Anti-sense 5′ CTTGGGAGCAAGAGGTGCT 3′
Catalase A (*CATA*)	Sense 5′ GCACAACGAAGTCCCTCAA 3′ Anti-sense 5′ CCGACATGGCCCACATAAA 3′
Mitochondrial peroxiredoxin (*PRX1*)	Sense 5′ CCTGCAGACAACCGATAAGA 3′ Anti-sense 5′ TCTTCACAGCAGGAGGAATG 3′
Peroxiredoxin (*HYR1*)	Sense 5′ AAGGCAAAGTCGTCCTCATC 3′ Anti-sense 5′ GTAGTCGAGAGGGAGGTGTAG 3′
S-transferase glutathione 2 (*GST2*)	Sense 5′ GAACCGCAAACCCTAATCCT 3′ Anti-sense 5′ GAGGAAGGTTGCGTAGTCTTTAT 3′

### Cell Extracts and Catalase Activity

aPb18 yeast cells, co-incubated or not with vEVs, and control vPb18 were submitted to oxidative stress in 10 mM H_2_O_2_ in Ham/glc for 2 h at 36°C, under agitation (170 rpm) in 24-well plates. Cell extracts were obtained by mechanical lysis of the cell pellets with an equal volume of glass beads in cold 10 mM Tris–HCl, pH 7.5 (700 ml/100 ml cell pellets) in vortex (5 cycles, 1 min each, 1 min cooling in ice in between). Whole cells and cell debris were respectively precipitated by centrifugation at 4,000×*g* for 15 min and two times at 15,000×*g* for 15 min at 4°C. The supernatants were protected from enzymatic proteolysis by the addition of protease inhibitors (EDTA-free Protease Inhibitor Cocktail—Sigma-Aldrich) and kept at −20°C until use. The protein content was estimated in cell extracts using the Bradford method (Bradford, 1976) adapted for microplates, with bovine serum albumin (BSA) as reference in standard curves. The catalase activity was estimated in triplicates following the Aebi (1984) methodology, also adapted for microplates. The reactions were monitored during 120 s with absorbance readings at 560 nm every 15 s. The results were calculated as activity/mg protein/s.

### Macrophage Stimulation With Pb18 EVs *In Vitro*

The effect of EV stimulation was analyzed *in vitro* using the Balb/C macrophage cell line RAW 264.7 and bone marrow-derived macrophages (BMDMs). The cells were maintained at 37°C under 5% CO_2_ in RPMI 1640 (Sigma-Aldrich, Burlington, MA, United States) supplemented with 10% fetal calf serum (FCS, Gibco, Carlsbad, CA, USA— South American Origin) and 100 mg/ml penicillin/streptomycin (RPMI/10% FCS). BMDM cells were obtained from male Balb/C femur and tibia bone marrow, as described by Zamboni and Rabinovitch (2003). The cells were differentiated by cultivating in RPMI/10% FCS supplemented with L929 fibroblast conditioned medium as the source of M-CSF (macrophage colony-stimulating factor). Differentiated BMDMs were maintained in RPMI 1640 supplemented with 2% FCS and 100 mg/ml penicillin/streptomycin at 37°C under 5% CO_2_ before the experiment. For the EV stimulation assays, RAW 264.7 (3 × 10^4^ cells) and BMDMs (1.5 × 10^5^ cells) were cultivated in 96-well plates for 24 h at 37°C under 5% CO_2_ and then co-incubated for 48 h with increasing amounts of vEVs and aEVs (1:10 up to 1:100,000 macrophage:EVs). Positive controls were stimulated with 1 μg/ml LPS. The expressed inflammatory mediators were quantified in the supernatants. The assays were repeated five times.

### Effect of EV Treatment Before *Paracoccidioides brasiliensis* Mice Infection

The effect of Pb18 EV inoculated in mice prior to fungal infection was evaluated using the PCM murine model (Taborda et al., 1998). Groups of 10 mice were inoculated subcutaneously with three doses (at 7-day intervals) of either vEVs or aEVs (1 × 10^8^ per dose) suspended in PBS (100 µl). Control groups received PBS alone. Fifteen days after the last dose, the animals were challenged by the intratracheal route with vPb18 (3 × 10^4^ viable yeast cells/50 µl PBS) and euthanized after 30 days of infection. PBS was used in non-infected controls. Lungs, liver, and spleen were excised for analysis and blood samples were collected by cardiac puncture for further analysis of inflammatory mediators. The collected organs were weighed, macerated in PBS, and plated in supplemented BHI medium. An aliquot of the macerated lungs was centrifuged at 2,500 rpm for 10 min at 4°C and the supernatant was kept at −20°C for further quantification of inflammatory mediators. Colony numbers were counted after 10 days of incubation at 37°C and recorded as colony-forming units/g of tissue (CFU/g). A small median-transversal section of each lung was excised before maceration, fixed in PBS/10% formalin for 24 h, dehydrated, and kept in 70% alcohol until they were embedded in paraffin for histopathology analysis. Five-micrometer lung sections were stained with hematoxylin–eosin and Grocott and observed using an Olympus BX-51 microscope. These experiments were carried out in biological duplicates.

### Quantification of Inflammatory Mediators

The analysis of inflammatory mediators was performed in EV-stimulated macrophages, serum samples, and lung extracts of infected mice. Cytokines (IL-6, IL-10, IL-12p70, IFN-γ, TNF-α) and chemokine MCP-1 were evaluated using the BD™ CBA Mouse Inflammation Kit (BD Biosciences, San Diego, CA, USA). The results were measured by cytometry in a FACSCanto II (BD) and analyzed by the FCAP Array v3 program. The quantification in pg/ml was based on standard curves built with the kit control cytokines. Nitric oxide (NO) levels were assayed following the Griess colorimetric method (Green et al., 1982). The results were evaluated spectrophotometrically at 540 nm and the concentrations were calculated by comparison with a dose–response standard curve of NaNO_2_.

### Statistical Analysis

The GraphPad Prism software (GraphPad Software Inc.) was used in all statistical analyses. The results were normalized by the D’Agostino and Pearson normality test and compared using the non-parametric one-way ANOVA test with multiple comparisons *via* the Tukey’s multiple comparisons and/or the Bonferroni’s multiple comparison tests. The results were plotted showing standard deviations as bars and considered significant for *p ≤*0.05.

## Results

### Characterization of *Paracoccidioides brasiliensis* EVs From vPb18 and aPb18

In order to compare the morphological and biological characteristics of vEVs and aEVs, respectively, from virulent vPb18 and its attenuated aPb18 variant, we isolated EVs from *P. brasiliensis* yeast cultures in agar Petri dishes following the basic strategy recently proposed by Reis et al. (2019). In previous works, we isolated EVs from fungal culture supernatants by filtration, concentration, and differential centrifugation (Rodrigues et al., 2007; Vallejo et al., 2011). The culture medium of choice for fungal growth and EV purification in our laboratory has been Ham/glc (Vallejo et al., 2011), although *Paracoccidioides* species grow better in rich media such as yeast–peptone–dextrose (YPD). We believe that cell culture defined media such as Ham’s F-12 may provide a closer host-like environment and fewer artifactual results eventually caused by complex molecules that might bind to the EV surface.

**Figure 1:**
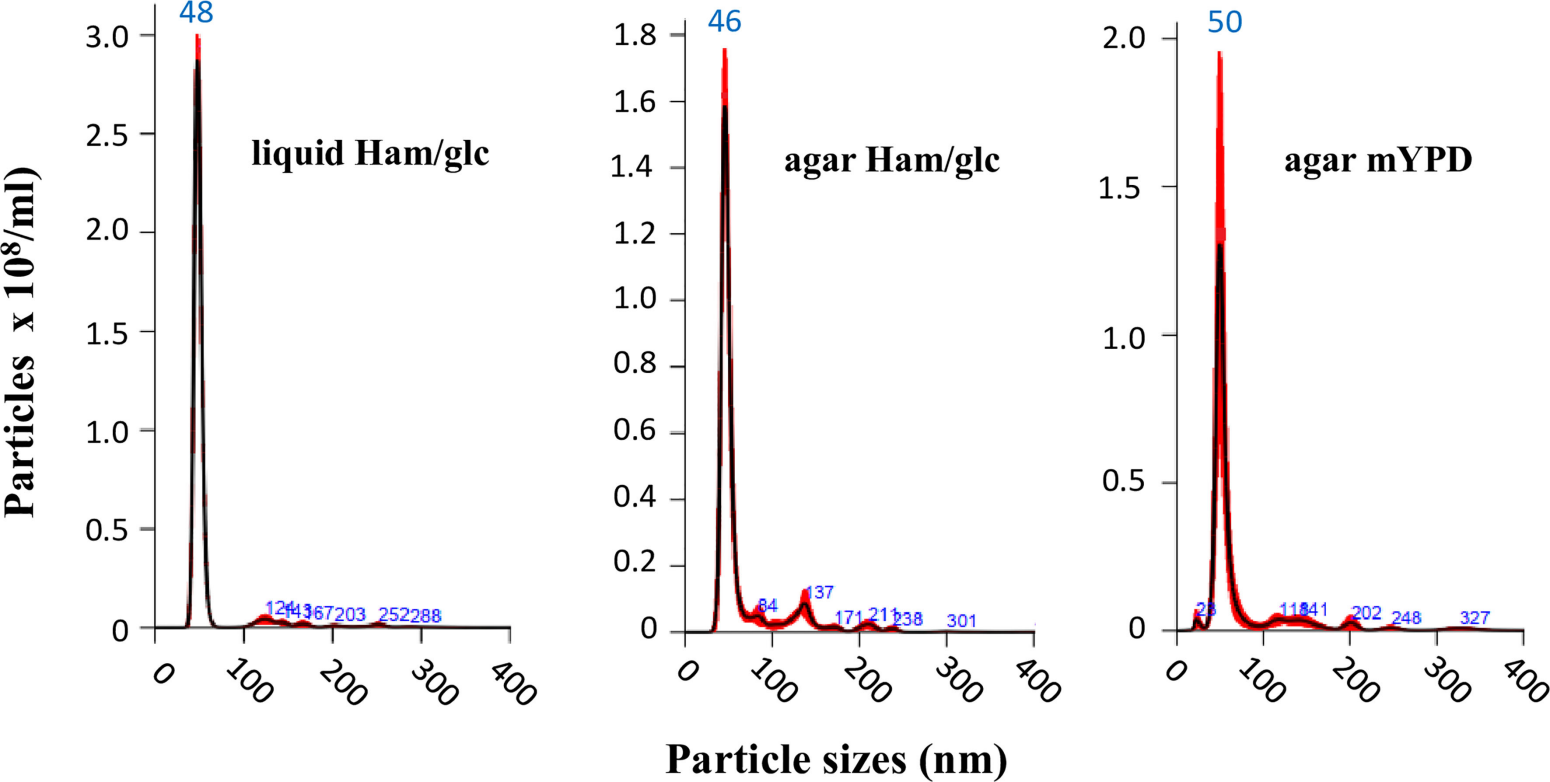
Comparative NTA analysis of extracellular vesicles (EVs) isolated from the yeast form of *Paracoccidioides brasiliensis* vPb18 cultivated in either liquid Ham/glc medium or Ham/glc and mYPD agar plates, as indicated. The graphics show the EV particle concentration (particles/ml) relative to the particle diameter (nm).

Nonetheless, we initially compared the results for yeasts cultivated in both defined Ham/glc (Vallejo et al., 2011) and complex mYPD agar media. This first step was carried out with the vPb18 isolate. By comparing dose–response assays, we found that plating 9 × 10^6^ yeast cells was sufficient to produce a confluent, over 95% viable growth after 2 days at 36°C. The NTA results in **Figure 1** show that the EVs isolated from vPb18 yeasts growing in agar plates and culture supernatants have similar size distribution, concentrating in a single peak of 46 to 50 nm diameter. Negligible amounts of EVs are distributed in discrete peaks between 80 and 300 nm. Therefore, the following experiments were carried out with EVs isolated from cell washes of vPb18 and aPb18 yeasts scrapped from confluent growth on Ham/glc agar plates.

**Figure 2:**
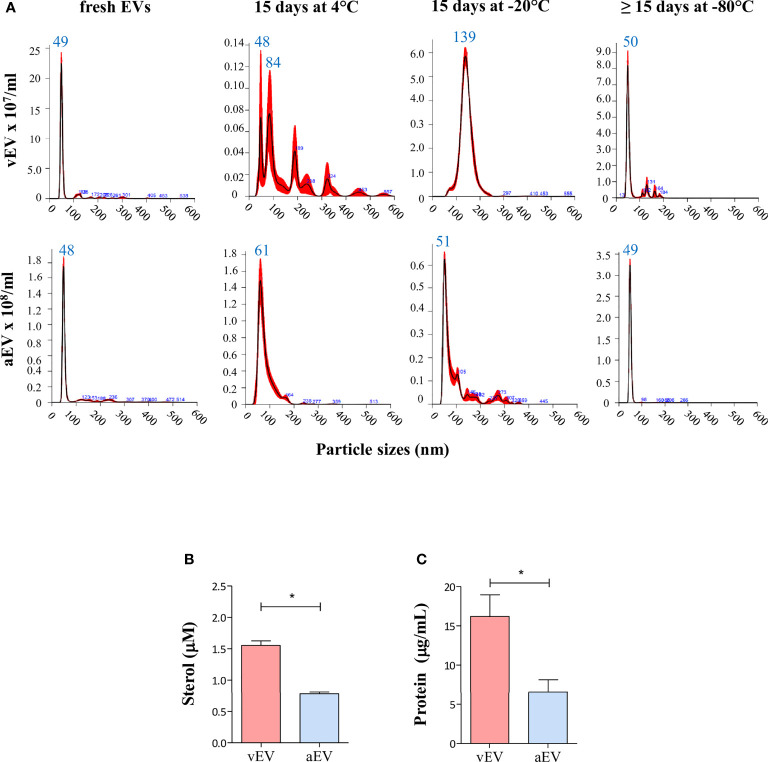
Comparative features of aEVs and vEVs. **(A)** NTA analysis of EVs isolated from the yeast form of *P. brasiliensis* vPb18 (vEVs, upper graphics) and its attenuated form aPb18 (aEVs, bottom graphics) cultivated in Ham/glc agar plates. The columns show the plots for the EV preparations assayed fresh (or up to 24 h at 4°C), after 15 days at 4°C, after 15 days at −20°C, and after 15 days or more at −80°C, as indicated. **(B, C)** Estimated **(B)** sterol (mM) and **(C)** protein (mg/ml) concentrations per 3 × 10^9^ EV particles. Statistically significant results are indicated as *p < 0.05.

Under those experimental conditions, freshly isolated vEVs and aEVs have similar size distribution between 30 and 300 nm, with about 80% of the EV particles sizing between 40 and 60 nm, but concentrated in a sharp peak of 48–49 nm (**Figure 2A**). Considering the average calculated from many preparations, we obtained a total of 1.3 × 10^10^ vEVs and 5.2 × 10^10^ aEVs per preparation (three plates). The estimated number of EVs/cell was 43 (vEVs) and 122 (aEVs). In terms of total sterol and protein contents, vEVs and aEVs also differed, both being significantly lower for aEV (**Figures 2B, C**).

While the size distribution profiles remained relatively similar for aEV samples recovered after storage for 15 days at 4°C, −20°C, or −80°C (median peaks between 48 and 61 nm), they considerably changed in stored vEV preparations at 4°C and −20°C (**Figure 2A**). After 15 days at 4°C, vEV peaks were 84, 189, 228, and 324 nm, and after 15 days at −20°C, we observed a broad peak between 100 and 200 nm (median 139 nm). After 15 days or more at −80°C, the vEV size profile was closer to that of fresh preparations (**Figure 2A**).

These results indicated that vEVs and aEVs have similar sizes in our experimental conditions, the yield of EVs exported per cell is higher for aEV than vEV, but the sterol and protein contents are lower for aEVs. In addition, the membrane surface characteristics are likely to vary, considering that vEVs apparently aggregated upon storage at 4°C and −20°C, while aEVs were relatively stable in all conditions. In further experiments, we used EVs that were either freshly isolated or stored at −80°C.

### Effect of vEVs on the aPb18 Phenotype

Antioxidant enzymes are more highly expressed in vPb18 than in aPb18, which in turn is more sensitive to growth under oxidative and nitrosative stress (Castilho et al., 2014; Castilho et al., 2018). On the other hand, vPb18 EVs carry proteins and RNA species related to stress (Vallejo et al., 2012b; Leitão, 2017; Peres da Silva et al., 2019). We therefore hypothesized that aPb18 co-incubated with vEVs could regain phenotypic antioxidant traits found in virulent vPb18. Our experimental design involved co-incubation of 1.5 × 10^10^ EVs with 1.5 × 10^8^ aPb18 yeast cells/ml for 4 h at 36°C, under agitation, based on previously established protocols (Leitão, 2017). The resulting cells were serially spotted for viability under stress in dose–response experiments. In a parallel experiment, the cells were oxidative-stressed in suspension and then tested for gene expression and enzyme activity.

**Figure 3:**
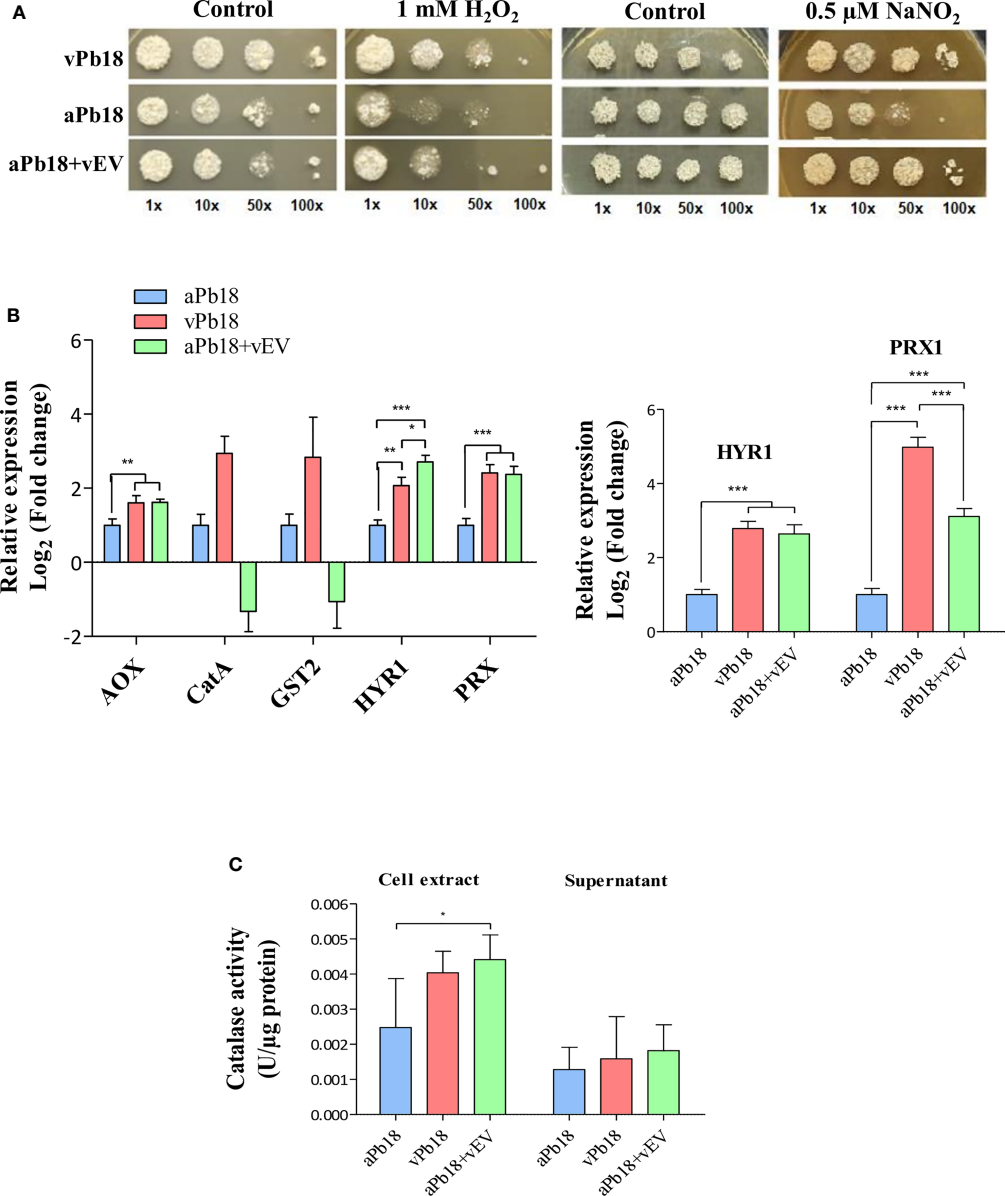
Effect of vEV co-incubation with aPb18. **(A)** aPb18 yeast cells co-incubated with vEVs (aPb18+vEVs) were evaluated for viability under stress in serial spot tests in mYPD plates supplemented with increasing concentrations of H_2_O_2_ (0, 0.1, 1, 5, and 10 mM) or NaNO_2_ (0, 0.25, 0.5, and 1 mM). The results for 1 mM H_2_O_2_ and 0.5 mM NaNO_2_ are shown, where the differences were more prominent. The plates were incubated for 7 days at 36°C and the images are representative of biological triplicates with technical duplicates. Serially spotted vPb18 and aPb18 were used as controls. **(B)** Gene expression of aPb18 yeast cells previously co-incubated with vEVs (aPb18+vEVs) was tested before (left graphic) or after (right graphic) a 2-h stress with 10 mM H_2_O_2_. Total fungal RNA was extracted and used in RT-qPCR of stress-related genes, as indicated. The α-tubulin gene was used as internal control in the comparative Ct strategy. The expression value for aPb18 cells was used as reference (1.0). **(C)** aPb18 yeast cells previously co-incubated with vEVs (aPb18+vEVs) were stressed for 2 h in 10 mM H_2_O_2_ and intracellular (cell extract) and secreted (supernatant) catalase activity was measured. vPb18 and aPb18 were used as controls in all experiments, which were carried out in triplicates. The standard deviations are shown with bars and statistically significant results are indicated as *p < 0.05, **p < 0.01, and ***p < 0.001.

**Figure 3A** shows that co-incubated aPb18+vEV cells had the same growth profile as vPb18 under the representative concentrations of 1 mM H_2_O_2_ and 0.5 µM NaNO_2_. The expression of antioxidant genes was evaluated in co-incubated aPb18+vEV cells submitted or not to oxidative stress for 2 h in 10 mM H_2_O_2_. Note that the constitutive expression profiles for the alternative oxidase *AOX* and peroxiredoxin *HYR1* and *PRX1* genes were significantly higher for vPb18 and aPb18+vEV cells than in aPb18 (**Figure 3B**). In stressed cells (right graphic), the *HYR1* and *PRX1* expression increased in both vPb18 and aPb18+vEV cells relatively to aPb18. The *CATA* and *GST2* (catalase A, glutathione transferase 2) transcripts were only detectable in non-stressed vPb18 (**Figure 3B**). The co-incubated aPb18v+EV cells had significantly more intracellular catalase activity than aPb18 after stress, reaching similar levels to those found in vPb18 (**Figure 3C**). The results for extracellular catalase activity were not statistically significant; however, the enzymatic activity tended to be higher in co-incubated aPb18vEV and vPb18 cells than in aPb18 after stress.

Overall, the results in **Figure 3** highly suggested that vEVs can induce phenotypic virulence traits related to stress that aPb18 lost after numerous passages in culture media, thus mimicking the effect obtained after animal passages in terms of gene and protein expression.

### Comparative Effect of vEV and aEV on Macrophage Stimulation *In Vitro*

**Figure 4:**
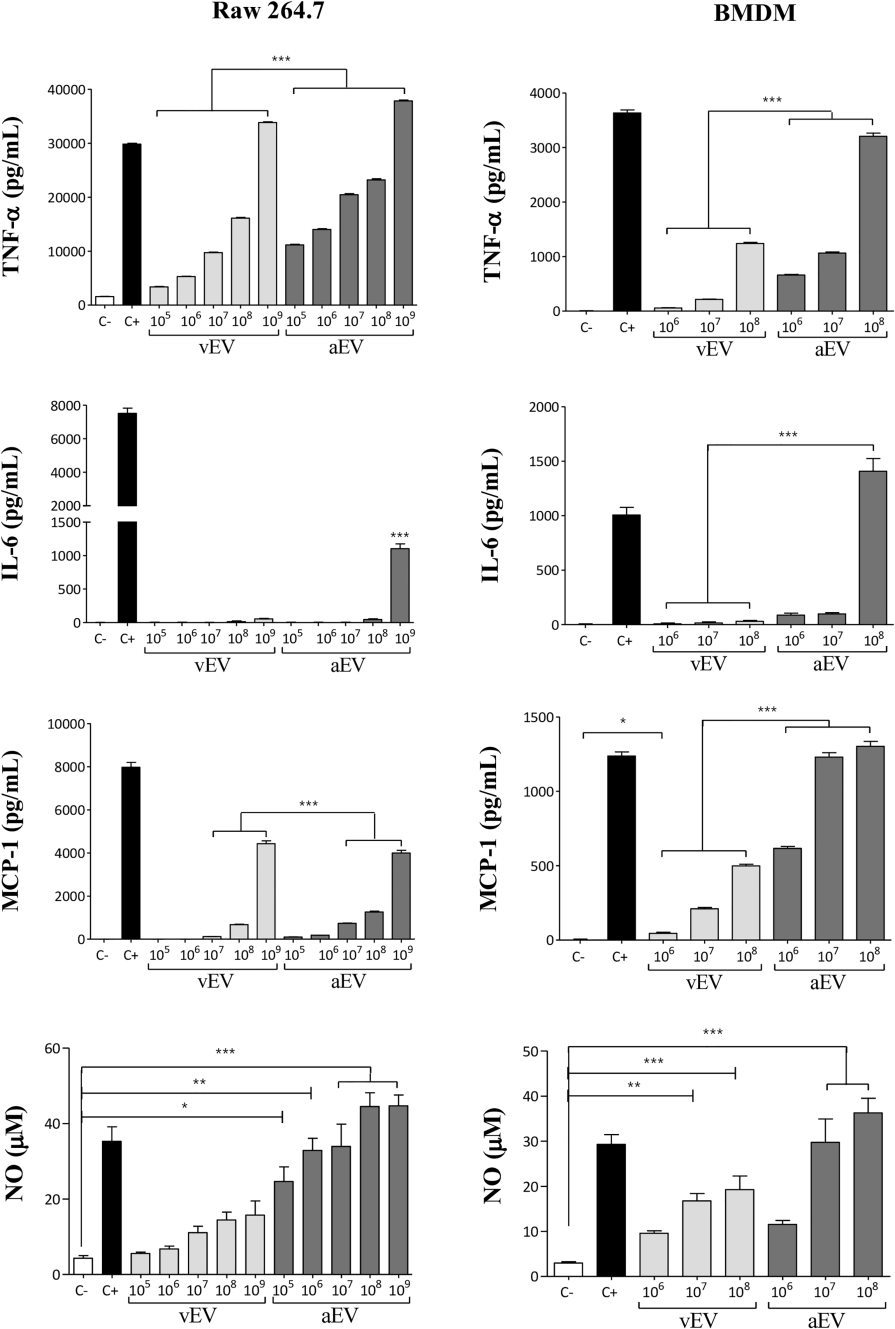
Comparative macrophage response upon stimulation with vEVs and aEVs. The graphics show the cytokine (TNF-α and IL-6), chemokine (MCP-1), and nitric oxide (NO) concentrations in culture supernatants of RAW 264.7 macrophages (left column) and BMDM (right column) co-incubated for 48 h with increasing concentrations of vEV or aEV, as indicated. C−, PBS (negative control); C+, 1 µg/ml LPS (positive control). Standard deviations are shown with bars and significant results are indicated as *p < 0.05, **p < 0.01, and ***p < 0.001.

Considering that vEVs and aEVs have peculiar characteristics and that vEVs were able to alter aPb18 phenotypic traits, we investigated if the *in-vitro* macrophage response would differ when stimulated with aEVs and vEVs. EV stimulation assays were carried out with non-activated macrophages of the RAW 264.7 cell culture and BMDM types from Balb/C mice. Dose–response experiments (**Figure 4**) showed that both vEVs and aEVs highly stimulated TNF-α expression; the levels were significantly higher upon aEV stimulus, especially at higher particle concentrations. The induction of IL-6 was detected only with higher concentrations of aEVs. In BMDM, chemokine MCP-1 was stimulated with aEVs at significantly higher levels than with vEVs. NO was also expressed at significantly higher levels after aEV than vEV stimulation. In our experimental conditions, pro-inflammatory IL-12 and anti-inflammatory IL-10 were only expressed in the positive controls and have not been detected even when macrophages were stimulated with high EV concentrations (not shown).

Overall, the results in **Figure 4** show that aEVs stimulate the expression of higher levels of pro-inflammatory TNF-α, IL-6, and NO *in vitro* than vEVs, suggesting that aEVs might have a role in the attenuated phenotype of the aPb18 variant. Chemokine MCP-1 was also higher after aEV stimulation.

### Comparative Effect of Treatment With vEV and aEV in Mice Infection

**Figure 5:**
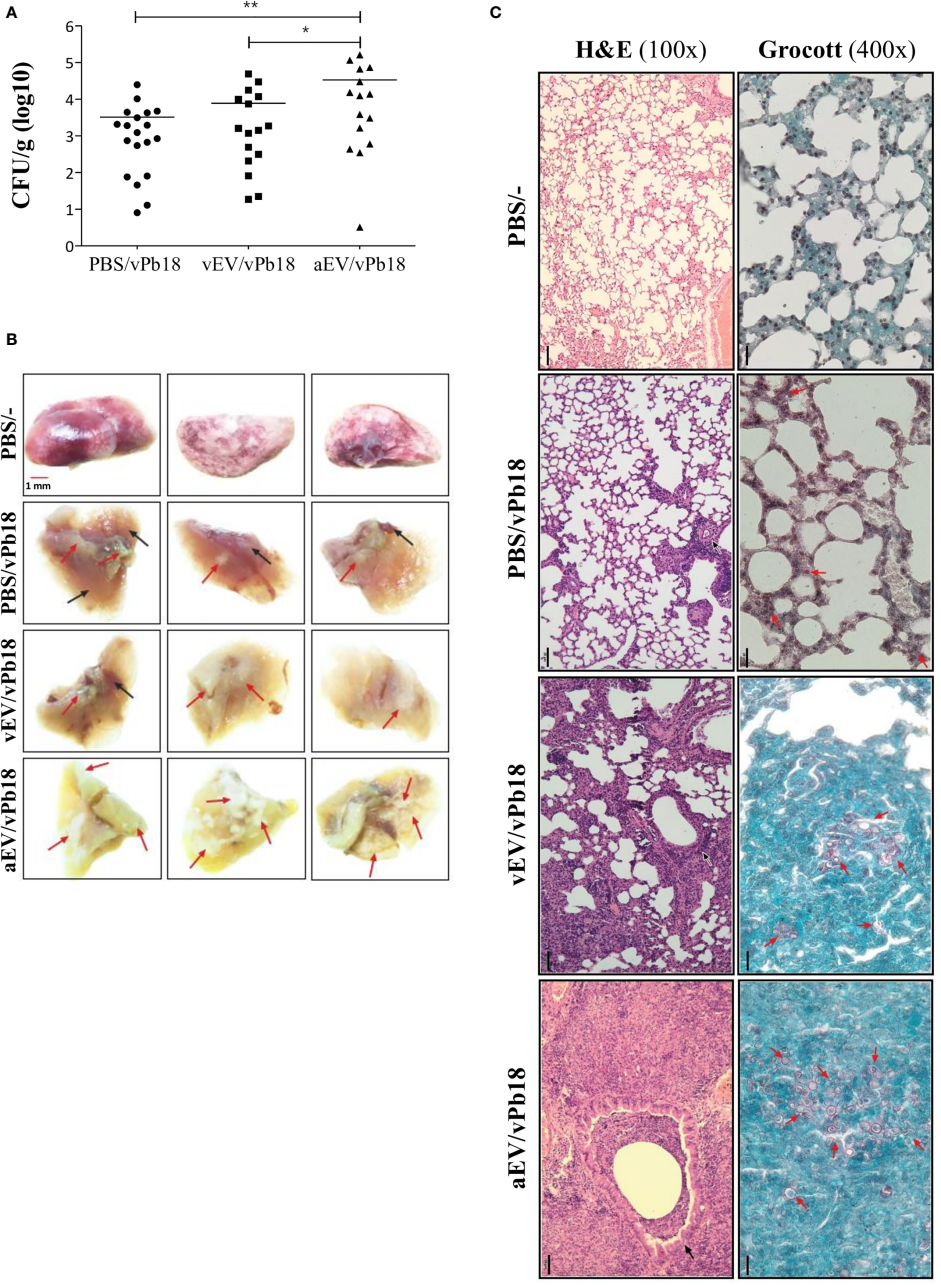
Comparative effect of vEV and aEV treatment before vPb18 challenge in the PCM murine model. Groups of 10 Balb/C mice were treated subcutaneously with three doses, 7-day intervals, of 1 × 10^8^ particles of vEV (vEV/vPb18) or aEV (aEV/vPb18) or with PBS (PBS/vPb18, positive control) before intratracheal infection with vPb18 (3 × 10^4^ cells) 14 days after the last dose. **(A)** Fungal load in the lungs after 30 days of infection recorded as colony-forming units (CFU/g of organ). Standard deviations are shown with bars and significant results are indicated as *p < 0.05 and **p < 0.01. **(B)** Macroscopy of representative lung samples from three different animals (columns), including non-infected controls (PBS/−). Black and red arrows point to, respectively, normal and damaged tissue. **(C)** Histopathology of lung sections stained with hematoxylin–eosin (H&E, ×100 magnification) or Grocott (×400 magnification), as indicated. Black bars correspond to 20 mm.

The high pro-inflammatory response stimulated by EVs *in vitro* suggested that aEVs might have a vaccination effect against PCM in mice. **Figure 5** shows that in our experimental conditions, both vEVs and aEVs exacerbated mice infection with vPb18 when analyzed in euthanized animals after 30 days post-challenge. We found that the fungal load in the lungs of the animals previously inoculated with aEVs was statistically higher than that in mice treated with vEVs (**Figure 5A**), while both groups had increased lung CFU than the control. Dissemination to the liver and spleen was negligible in all samples (not shown). However, 20% of the animals in the EV-treated groups unexpectedly died before 30 days (not shown).

Macroscopically, the lungs of the treated animals looked sick, with pale color due to extensive areas of inflammation and fibrosis (**Figure 5B**). They had a hard texture, as opposed to the soft touch of the negative controls and the partially affected positive controls. Microscopically, the lung architecture was lost in the animals previously treated with EVs (vEV/vPb18, aEV/vPb18), the interalveolar septa were widened and diffused, and non-organized granulomas were filled with yeasts (**Figure 5C**). The microscopic aspect was more dramatic for the animals previously treated with aEVs than with vEVs, presenting with hemorrhagic spots and numerous fungal structures. The positive controls had most of the lung architecture preserved, but small granulomas involving a few yeast cells can be seen (**Figure 5C**, PBS/vPb18).

**Figure 6:**
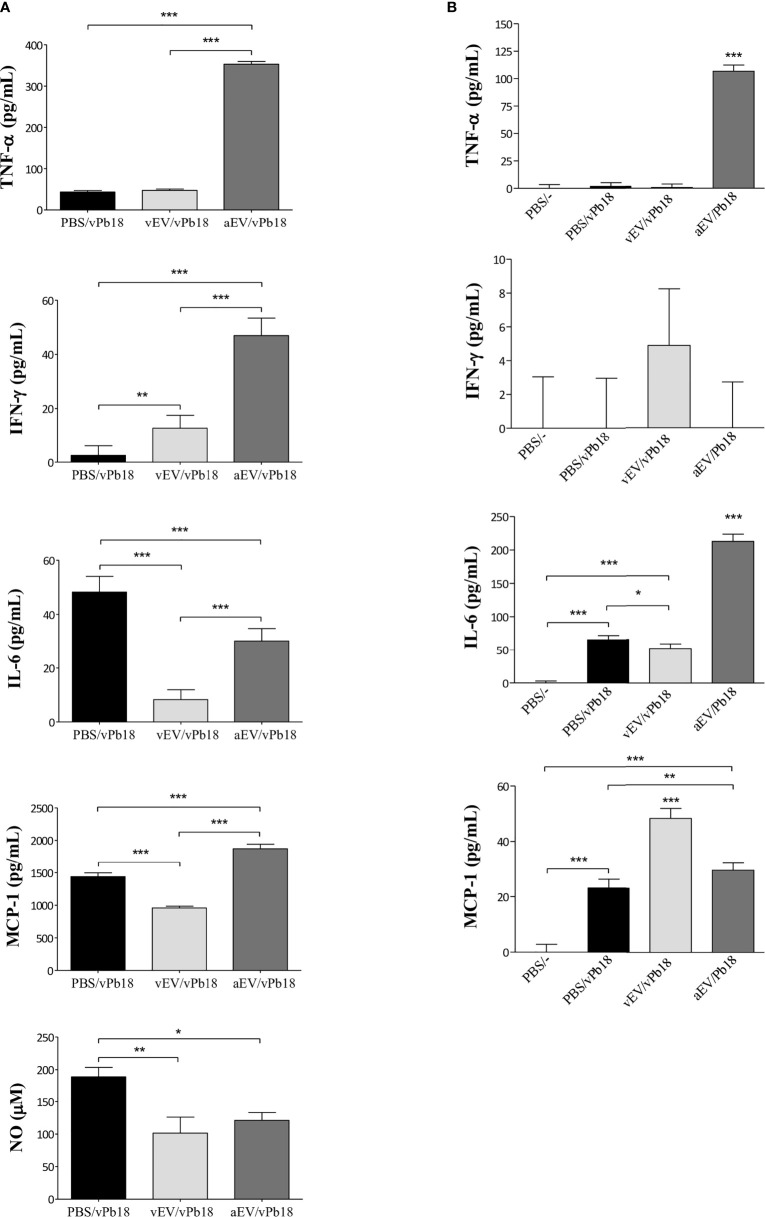
Inflammatory mediators estimated in mice inoculated with either vEVs (vEV/vPb18) or aEVs (aEV/vPb18) before challenge with *P. brasiliensis* vPb18, as detailed in **Figure 4**. **(A)** Lung TNF-α, INF-γ, IL-6, and MCP-1, and NO concentrations. **(B)** TNF-α, INF-γ, IL-6, and MCP-1 concentrations; PBS/−, non-infected animals. Standard deviations are shown with bars and significant results are indicated as *p < 0.05, **p < 0.01, and ***p < 0.001.

We estimated the concentration of inflammatory mediators in lung extracts (**Figure 6A**) and circulating in mice sera (**Figure 6B**). We found that the lung TNF-α, IFN-γ, and MCP-1 concentrations were specially increased in aEV/vPb18 animals when compared with non-treated controls and vEV/vPb18. The level of lung NO was comparable in the treated samples and significantly lower than in the untreated controls. Lung IL-6 levels were also decreased in EV-treated animals in comparison with the controls, but significantly higher in aEV/vPb18 than in vEV/vPb18. The profiles for circulating IL-6, IFN-γ, and MCP-1 were diverse, since IFN-γ and MCP-1 were statistically higher in the vEV/vPb18 group, while IL-6 was higher in the aEV/vPb18 group. Pro-inflammatory IL-12 and anti-inflammatory IL-10 were only detected in the positive LPS controls.

Together, the results in **Figures 5** and **6** show that, in our experimental conditions, subcutaneous treatment with both vEVs and aEVs previously to fungal challenge exacerbated murine PCM; however, the effect with aEV was much more pronounced, probably due to a hyperinflammatory response suggested by the profiles of inflammatory mediators.

## Discussion

The virulent/attenuated Pb18 model of *P. brasiliensis* proved to be useful to study the roles of fungal EVs because it consists of variants from the same isolate transiently displaying higher or attenuated virulence capacity in mice. We have been able to improve the resistance of attenuated aPb18 to grow under oxidative and nitrosative stress upon previous incubation with vEVs from virulent vPb18. We showed that aEVs from the attenuated aPb18 variant generally stimulated the expression of higher levels of inflammatory mediators both *in vivo* and *in vitro*, which probably contributed to the exacerbated *P. brasiliensis* murine infection we observed in aEV-treated animals.

Tolerance to oxidative stress is an important virulence factor in microorganisms (Kaihami et al., 2014; Rocha et al., 2018), and proteins involved in the oxidative stress response in *P. brasiliensis* yeast cells tend to be upregulated during macrophage infection and under laboratory stress conditions (Batista et al., 2007; Parente-Rocha et al., 2015; Castilho et al., 2018). In our work, the stress resistance trait acquired by vEV-treated aPb18 paralleled the higher expression pattern of the alternative oxidase *AOX* and peroxiredoxin *HYR1* and *PRX1* genes and of the catalase activity. We have recently reported that PbPrx1 is a peroxidase distributed in the cytosol, mitochondria, cell wall, and extracellular vesicles of *P. brasiliensis* cells (Vallejo et al., 2012b; Longo et al., 2020). PbPrx1 is highly induced by, and reactive with, organic hydroperoxides, thus suggesting an important role in fungal protection against these effector molecules.

The effect of vEVs in the phenotypic change of attenuated aPb18 reported here brings relevant contribution to the fungal EV literature, although aspects like the mechanism(s) involved and the virulence *in vivo* of vEV-treated aPb18 yeast cells are yet to be explored by our group. The evidence that EVs can cross the cell wall inwards (Walker et al., 2018) and signal fungal cells is only starting to be demonstrated. Bielska et al. (2018) showed that EVs from virulent *Cryptococcus gattii* are internalized by infected macrophages and affect the growth rate of dormant yeasts, apparently by transference of regulatory small RNA species to the attenuated fungal cells inside the macrophages.

We optimized the protocol of EV production of *Paracoccidioides* yeast cell washes from confluent cultures in Ham’s F-12 agar medium, based on the strategy recently proposed for *C. neoformans* (Reis et al., 2019; Reis et al., 2021). *Paracoccidioides brasiliensis* cells grow slowly and the EVs they produce seem to be smaller than those from other pathogenic yeasts (Reis et al., 2019; Vargas et al., 2020; Reis et al., 2021). In our experimental conditions, however, *P. brasiliensis* Pb18 yeasts apparently exported considerably more EVs per cell than *Cryptococcus* spp. [43 to 122 here × 0.4 to 5 EVs/cell in Reis et al. (2021)]. We observed that we can store both vEVs and aEVs at −80°C for later use, considering that the EV sizes remain about the same as those found in fresh preparations. Storage at 4°C and −20°C, on the other hand, highly affected vEVs by increasing their sizes, probably due to aggregation. Since that effect was only slightly seen in aEVs, this difference suggests that the surface composition differs in aEVs. Vargas et al. (2020) observed that *C. albicans* EVs were generally stable after 7 days at 4°C, −20°C, and −80°C. However, −80°C seemed to be the best storage option since the EV sizes were even closer to those of freshly prepared EVs. Additionally, the capacity to stimulate cytokine and protect *Galleria mellonella* from *Candida* infection was preserved after storage at −80°C, in spite of being decreased.

Our results of macrophage EV stimulation are comparable to those reported by da Silva et al. (2016) in the sense that we also showed that EVs from Pb18 stimulated pro-inflammatory mediators in a dose-dependent manner. The originality of our work relies on the demonstration that aEVs from attenuated aPb18 stimulated significantly higher expression of macrophage inflammatory mediators. The stimulation of high levels of pro-inflammatory mediators seen here suggested that aEVs would contribute to a more efficient fungal elimination, in concert with the poor infectivity of aPb18 in mice. Surprisingly, however, in our experimental conditions, neither vEVs nor aEVs protected against murine PCM. Our results substantially differ from those recently published by Baltazar et al. (2021), who reported a vaccination effect of Pb18 EVs in C57BL/6 mice, as analyzed 14 and 72 days after challenge with Pb18. The conflicting results are probably due to differences in experimental conditions. The animal model (Balb/C versus C57BL/6), the number of EV boosters, the use of adjuvant, and the length of infection were distinct. Most importantly, we isolated EVs from *P. brasiliensis* Pb18 cultivated in defined agar Ham’s F-12 medium, as opposed to the agitated cultures in YPD-rich medium used by Baltazar et al. (2021). Culture conditions ultimately determine the microorganism transcription responses, the arsenal of expressed molecules, and hence, the level of host–fungi interaction. The EV cargo is likely to reflect this scenario, as experimentally demonstrated for *H. capsulatum* EVs isolated from fungal yeast cells grown in complex BHI versus defined RPMI and Ham’s F-12 cell culture media (Cleare et al., 2020).

Additionally, the current fungal EV literature results on the protective role of EVs *in vivo* can vary with the fungal model and the experimental protocols: while fungal EVs were able to vaccinate mice against *C. albicans* and *C. neoformans* infection (Vargas et al., 2015; Vargas et al., 2020; Rizzo et al., 2021), they enhanced subcutaneous infection with *Sporothrix brasiliensis* in mice (Ikeda et al., 2018). In the cryptococcosis model, the mortality of mice treated with fungal EV-stimulated macrophages increased upon infection with *C. neoformans* (Zhang et al., 2021). Cryptococcal EVs also facilitated the fungal crossing to the brain and enhanced meningoencephalitis (Huang et al., 2012). The enhancement of murine sporotrichosis by treatment with fungal EVs was attributed to a hyperinflammatory reaction (Ikeda et al., 2018). That explanation seems to apply to the present work, notably to the effect of aEVs from attenuated *P. brasiliensis* Pb18. The level of lung and circulating TNF-α and IFN-γ was over five-fold higher for aEV- than for vEV-treated or control mice after 30 days of infection. The lung NO concentration, on the contrary, was half the levels found in control mice, which probably stimulated fungal growth instead of eliminating the yeasts, as observed to happen *in vitro* for *P. brasiliensis* cultured in low concentrations of NO (Haniu et al., 2013). Differences in the vEV and aEV cargo, especially at the membrane surface, are likely to explain the present results. Rizzo et al. (2021) obtained partial protection in preliminary experiments based on adjuvant-free intraperitoneal mice inoculations of cryptococcal EVs from an acapsular mutant and the dose was calculated as protein concentration. Vargas et al. (2020) obtained 100% survival of immunosuppressed mice infected with *C. albicans* using three doses (as sterol concentration) of EVs alone or inoculated with adjuvant *via* the intraperitoneal route. Ikeda et al. (2018) tested two doses of 8 × 10^7^ and 3.5 × 10^8^ *S. brasiliensis* EVs inoculated *via* the intramuscular route, which induced exacerbation of subcutaneous sporotrichosis. Baltazar et al. (2021) managed to reduce the *P. brasiliensis* burden of mice treated with fungal EVs *via* the subcutaneous route using adjuvant in the first dose (calculated as protein concentration).

Fungal EVs carry antigens, virulence factors, and molecules that stimulate the innate immune system among a blend of proteins, polysaccharides, oligosaccharides, active enzymes, small regulatory RNA, pigments, and other still less explored components (Zamith-Miranda et al., 2021b). The number and variety of the reported fungal EV cargo has increased lately with the interest to study these structures. EVs are likely to participate in cell wall remodeling (Longo et al., 2014; Nimrichter et al., 2016; Zhao et al., 2019; reviewed in Rizzo et al., 2020) as well as in the synthesis of *Candida* biofilms (Zarnowski et al., 2018; Zarnowski et al., 2021). They participate in the synthesis of the main virulence factor in *Cryptococcus*, which is the high molecular weight glucuronoxylomannose component of the capsule (Rodrigues et al., 2008; Reis et al., 2019; reviewed in de Oliveira et al., 2020; Rizzo et al., 2020). The presence of peptides has been recently reported in *Cryptococcus* EVs, and one of them proved to protect against infection in *G. mellonella* (Reis et al., 2021). However, we are only starting to understand the variables involved in the biogenesis of the EV cargo and the scope of interaction of fungal EVs with the host and fungal cells. In that sense, our work brings an important contribution to the understanding of the role of fungal EVs in the communication with fungal cells and with the host. It is clear that the effect of EVs in fungal infection depends on the established experimental conditions because EVs are complex and dynamic structures. Therefore, EV protection protocols have to be carefully tested.

## Data Availability Statement

The original contributions presented in the study are included in the article/supplementary material. Further inquiries can be directed to the corresponding author.

## Ethics Statement

The animal procedures followed the ethical handling of laboratory animals approved by the UNIFESP Animal Experimentation Ethics Committee (CEUA/UNIFESP), under protocol number 8450221018.

## Author Contributions

CO designed and performed the experiments. NA performed specific enzymatic experiments. RP designed the experiments and wrote the manuscript. All authors critically reviewed and approved the final manuscript.

## Funding

We thank Conselho Nacional de Desenvolvimento Científico e Tecnológico -CNPq (CNPq Productivity Award PQ 313375/2017-8 and NEA scholarship) and Coordenação de Aperfeiçoamento de Pessoal de Nível Superior - CAPES (CEO scholarship) for financial support. We also thank the support provided by the grant FAPESP 2013-25950-1.

## Conflict of Interest

The authors declare that the research was conducted in the absence of any commercial or financial relationships that could be construed as a potential conflict of interest.

## Publisher’s Note

All claims expressed in this article are solely those of the authors and do not necessarily represent those of their affiliated organizations, or those of the publisher, the editors and the reviewers. Any product that may be evaluated in this article, or claim that may be made by its manufacturer, is not guaranteed or endorsed by the publisher.
